# Plain Talks on Avoided Subjects

**Published:** 1883-01

**Authors:** 


Plain Talks on Avoided Subjects. By H. N. Guernsey, M. D. Price, $1.00. M. A. Davis: Philadelphia. 1882.

In this little monograph Dr. Guernsey gives some sensible advice to the laity—for whom we presume the book is intended.

